# Comparison of Simultaneous Nitrification and Denitrification for Three Different Reactors

**DOI:** 10.1155/2015/901508

**Published:** 2015-08-03

**Authors:** W. Khanitchaidecha, A. Nakaruk, P. Koshy, K. Futaba

**Affiliations:** ^1^Department of Civil Engineering, Faculty of Engineering, Naresuan University, Thailand; ^2^Centre of Excellence for Innovation and Technology for Water Treatment, Naresuan University, Thailand; ^3^Department of Industrial Engineering, Faculty of Engineering, Naresuan University, Thailand; ^4^Center of Excellence for Environmental Health and Toxicology, Naresuan University, Thailand; ^5^School of Materials Science and Engineering, The University of New South Wales, Australia; ^6^International Research Centre for River Basin Environment, University of Yamanashi, Japan

## Abstract

Discharge of high NH_4_-N containing wastewater into water bodies has become a critical and serious issue due to its negative impact on water and environmental quality. In this research, the performance of three different reactors was assessed and compared with regard to the removal of NH_4_-N from wastewater. The highest nitrogen removal efficiency of 98.3% was found when the entrapped sludge reactor (ESR), in which the sludge was entrapped in polyethylene glycol polymer, was used. Under intermittent aeration, nitrification and denitrification occurred simultaneously in the aerobic and anaerobic periods. Moreover, internal carbon was consumed efficiently for denitrification. On the other hand, internal carbon consumption was not found to occur in the suspended sludge reactor (SSR) and the mixed sludge reactor (MSR) and this resulted in nitrogen removal efficiencies of SSR and MSR being 64.7 and 45.1%, respectively. Nitrification and denitrification were the main nitrogen removal processes in the aerobic and anaerobic periods, respectively. However, due to the absence of sufficient organic carbon, denitrification was uncompleted resulting in high NO_3_-N contents in the effluent.

## 1. Introduction

Many countries are currently facing serious issues related to drinking water quality due to increasing water pollution which has resulted in a rapid growth of aquatic plants. This phenomenon is known as eutrophication results from the discharge of wastewater, which contains high amounts of nitrogen-containing species, from households and agricultural sources [1, 2]. Ammonium-nitrogen (NH_4_-N) and nitrate-nitrogen (NO_3_-N) are two main forms of nitrogen-containing species present commonly in wastewater. Previous studies by others [3–5] have shown that high NH_4_-N concentrations (40–50 and 30–40 mg/L) are seen in municipal and agricultural wastewaters, respectively. These levels are considerably greater than the acceptable levels of NH_4_-N and NO_3_-N, which are 0.5 and 5 mg/L, respectively [6]. With increasing global population and greater demands on agriculture, there has been a corresponding increase in the volumes of wastewaters containing high amounts of NH_4_-N.

To combat the problem of eutrophication, England and Wales governments have spent in large amounts of money ($77 million annually) to clean up affected water sources [7]. However, these costs can be reduced significantly by treating the problem at its sources, that is, by treating the waste water effectively prior to its discharge in larger water bodies.

Biological treatment is a widely used technology for pilot-scale nitrogen removal from wastewater. This technique is effective owing to its low cost and high treatment capacity, compared to more advanced treatment technologies such as membrane and electrocoagulation [3, 8, 9]. Biological nitrogen treatment involves processes such as nitrification, denitrification, simultaneous nitrification and denitrification (SND), single-reactor system for high-activity ammonium removal over nitrite (SHARON), and anaerobic ammonium oxidation (Anammox), as summarised in Table 1. Among these mentioned processes, only SND and Anammox are capable of effecting almost complete NH_4_-N removal while not generating other toxic nitrogen forms such as nitrite-nitrogen (NO_2_-N) and nitrate-nitrogen (NO_3_-N).

Significant limitations of Anammox system include their sensitivity to oxygen and low growth rate of the resident microorganisms. Moreover, a pretreatment system is required to oxidise NH_4_-N to NO_2_-N in the case of SHARON [10, 11]. On the other hand, the SND system can complete NH_4_-N removal in a single reactor and is easy to operate [12]. The SND system has been operated in a sequencing batch reactor which use suspended sludge and has typically the following cycle of operations: water inflow, aerobic (4.0–7.0 h), anaerobic (3.0-4.0 h), settling (0.5–1.0 h), and water drainage [13, 14]. However, the proposed sequencing batch reactor requires a long treatment period.

In the meantime, the advantage of using entrapped sludge for nitrogen wastewater treatment has been suggested, such as tolerating inhibiting environment and enhancing nitrogen removal efficiency [15]. The aim of this research is to compare the performance of various reactors consisting of suspended and/or entrapped sludge for NH_4_-N removal via the SND process. In this respect, the reactors were operated at different air flow rates, air supply periods, and carbon/nitrogen (C/N) ratios to determine the mechanisms inside the reactors.

## 2. Methodology

### 2.1. Reactor Setup and Operation

**Figure 1:**
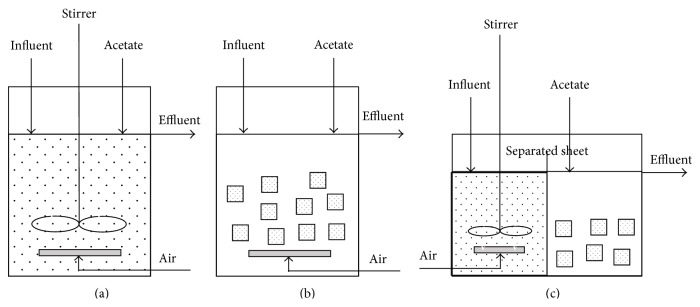
Schematic diagrams of (a) suspended sludge reactor (SSR), (b) entrapped sludge reactor (ESR), and (c) mixed sludge reactor (MSR).

Three reactors—(a) suspended sludge reactor (SSR), (b) entrapped sludge reactor (ESR), and (c) mixed sludge reactor (MSR)—were designed and used. The schematic diagrams and operating conditions for all reactors are summarised in Figure 1 and Table 2, respectively.

For the SSR, the acclimatised sludge was added to the cylindrical reactor; the initial sludge concentration was approximately 3500 mg/L. The influent contained ~40–50 mg/L of NH_4_-N and other necessary substrates such as HCO_3_^−^, PO_4_^3−^, and Mg^2+^ [13]. The NO_2_-N and NO_3_-N levels in the influent were <3 mg/L. The air was supplied from the bottom of the reactor for two hours (aerobic) and then air supply was ceased for the next two hours (anaerobic); this intermittent supply of air was continued till the end of the operation (approximately 24 hours). A 300 mg/L of acetate solution was prepared and added once at the time of the first anaerobic reaction to maintain the C/N ratio at 1.5.

For the ESR, the acclimatised sludge was entrapped in polyethylene glycol polymer, and then the sludge gel was cut into 3 × 3 × 3 mm pellets. The sludge pellet was added to the cylindrical reactor to fill up ~25% of the reactor volume. The influent NH_4_-N, air supply, and C/N levels were controlled as the previous reactor.

For the MSR, the rectangular reactor was separated into two chambers: the acclimatised sludge was added in the first chamber and the sludge pellet was added in the second chamber. The water was passed from the first chamber to the second chamber via a separated sheet. The influent and air were supplied continuously to the first chamber (aerobic) and the acetate was fed to the second chamber (anaerobic). The influent NH_4_-N and C/N levels were controlled as in the case of the previous reactors.

### 2.2. Nitrification and Denitrification Tests

To determine the nitrification rate, the NH_4_-N water was fed into the reactors operating under continuous air supply with no acetate addition. The water samples were collected every hour for six hours to analyse the NH_4_-N, NO_2_-N, and NO_3_-N concentrations. For determining the denitrification rate, the NO_3_-N water was fed into the reactors and acetate was also added at the C/N of 1.5. Air was not supplied and the dissolved oxygen (DO) amount was <0.5 mg/L during the tests. The water samples were collected every hour for six hours to analyse the NH_4_-N, NO_2_-N, NO_3_-N, and carbon concentrations.

### 2.3. Water Quality Analysis

The concentrations of NH_4_-N, NO_2_-N, and NO_3_-N in influent and effluent were measured using phenate, colorimetric, and ultraviolet spectrophotometric screening methods in accordance with the standard methods used for the examination of water and wastewater [16]. The organic carbon was determined as chemical oxygen demand (COD) concentration using COD analyser (AL200 COD VARIO Photometer). The reactor performance, nitrification rate, and denitrification rate were calculated using (1)–(3):

(1) N  removal  efficiency=1−NH4-Neff+NO2-Neff+NO3-NeffNH4-Ninf×100

(2) $$\begin{array}{c} Nitrification\;rate=\frac{{\left[{NH}_{4}\text{-}N\right]}_{0}-{\left[{NH}_{4}\text{-}N\right]}_{t}}{t} \end{array}$$

(3) $$\begin{array}{c} Denitrification\;rate=\frac{{\left[{NO}_{3}\text{-}N\right]}_{0}-{\left[{NO}_{2}\text{-}N\right]}_{t}-{\left[{NO}_{3}\text{-}N\right]}_{t}}{t}, \end{array}$$

where [NH_4_-N]_inf_ and [NH_4_-N]_eff_ = NH_4_-N concentrations (mg/L) in influent and effluent, [NO_3_-N]_eff_ = NO_3_-N concentration in the effluent (mg/L), [NO_2_-N]_eff_ = NO_2_-N concentration in the effluent (mg/L), [NH_4_-N]_0_ and [NH_4_-N]_*t*_ = NH_4_-N concentration at time 0 and *t* (mg/L), [NO_3_-N]_0_ and [NO_3_-N]_*t*_ = NO_3_-N concentration at time 0 and *t* (mg/L), [NO_2_-N]_*t*_ = NO_2_-N concentration at time *t* (mg/L), and *t* = time (h).

## 3. Results and Discussion

### 3.1. Performance of Suspended Sludge Reactor

**Figure 2:**
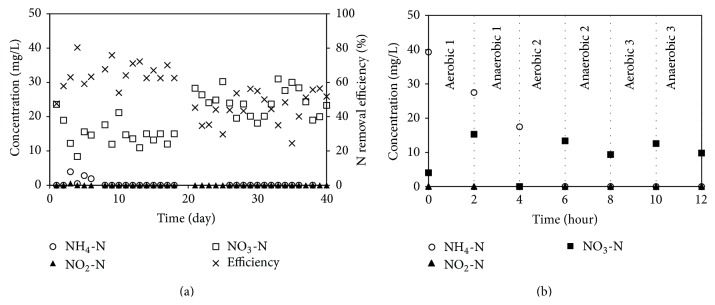
(a) Nitrogen removal efficiency and (b) nitrogen profile of SSR.

The SSR was operated under intermittent aeration for two hours. In aerobic conditions, a low air flow rate of 0.5 L/min was used and the DO was ~4-5 mg/L. In the anaerobic setup, the air was not supplied and this caused the DO to drop immediately to ~0.5 mg/L within 30 min. The nitrogen removal efficiency and nitrogen concentrations during operation are presented in Figure 2(a) (for days 1–20). The nitrogen removal efficiency was 64.7% and the 15 mg/L of NO_3_-N remained in the effluent. These results reveal that the nitrification process to change NH_4_-N to NO_2_-N and NO_3_-N occurred to completion. However, the denitrification process to change NO_3_-N to N_2_ was ineffective. From previous studies [14, 17], it is suggested that the ineffective denitrification occurs due to high oxygen, high NO_2_-N, and low organic carbon contents. The nitrogen profile during the three cycles of intermittent aeration (including aerobic 1, anaerobic 1, aerobic 2, anaerobic 2, aerobic 3, and anaerobic 3) was determined to identify the main cause of ineffective denitrification (Figure 2(b)). After addition of the acetate during anaerobic 1 step, the NO_3_-N level decreased from 16 mg/L to zero and the COD concentration also decreased from 200 to 8 mg/L. The COD concentration was stable at ~5–8 mg/L till the end of the operation and there was no NO_3_-N reduction in anaerobic 2 and 3 stages. Since the NO_2_-N was not detected and the DO was also low as 0.5 mg/L in the anaerobic stage, thus the incomplete denitrification in the SSR resulted from insufficient acetate addition (C/N of 1.5).

From Figure 2(b), the main nitrogen removal processes that occurred in the SSR were nitrification and denitrification. Nitrification was the dominant process in aerobic 1 stage when NH_4_-N and O_2_ contents were high. In the anaerobic 1 stage, simultaneous nitrification and denitrification occurred, as seen from the supporting data of decreasing NH_4_-N and NO_3_-N contents. The excess NO_3_-N and organic carbon contents induced the occurrence of denitrification. Moreover, the NH_4_-N was oxidised using the remaining oxygen from aerobic 1 stage. The NH_4_-N was oxidised continuously in aerobic 2 stage, until its concentration became zero. However due to lack of sufficient organic carbon (~5 mg/L of COD remained), the NO_3_-N still remained in the effluent.

The rates of nitrification and denitrification were estimated (not shown here). The results are summarised in Table 3; the denitrification rate of 1.8 mg/L-min was much faster than the nitrification rate of 0.2 mg/L-min under this condition. Since the nitrification rate can be increased with increasing DO concentration [18], the SSR was operated continuously at a higher air flow rate of 1 L/min (days 21–40 in Figure 2(a)). At the high air flow rate, the nitrification rate was increased from 0.2 to 0.3 mg/L-min; however the denitrification rate and nitrogen removal efficiency decreased to 1.6 mg/L-min and 44.9%, respectively. This is because the high air flow rate led to high oxygen retention in the anaerobic stage resulting in decreasing denitrification activity. Thus it can be concluded that the SSR can achieve the highest efficiency of 64.7% for a nitrification rate of 0.2 mg/L-min and a denitrification rate of 1.8 mg/L-min. To improve the reactor performance, a high C/N ratio > 1.5 should be maintained.

### 3.2. Performance of Entrapped Sludge Reactor

**Figure 3:**
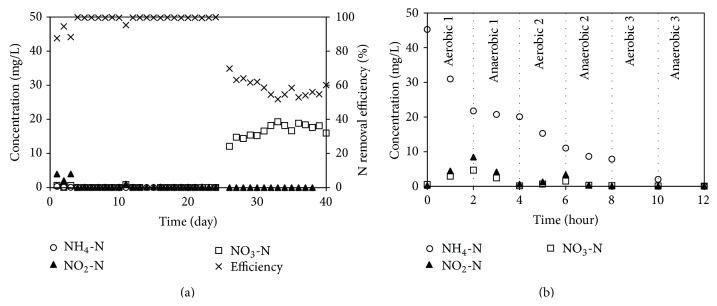
(a) Nitrogen removal efficiency and (b) nitrogen profile of ESR.

The ESR was operated under intermittent aeration for two hours; the DO was ~4-5 mg/L in aerobic condition and this value dropped to ~0.5 mg/L in anaerobic condition. During 25 days of operation, the variations in the nitrogen removal efficiency and nitrogen concentrations are presented in Figure 3(a). The nitrogen removal efficiency was relatively stable at 98.3%, even though a low C/N ratio of 1.5 was maintained as was the case in the SSR. In the aerobic 1 stage in Figure 3(b), the 24 mg/L of NH_4_-N was oxidised while only 13 mg/L of NO_2_-N and NO_3_-N was detected. These results suggest that nitrification and denitrification occurred simultaneously in aerobic condition which had bulk DO value of ~4-5 mg/L. Due to the mass transfer (of oxygen, NH_4_-N, and NO_3_-N) inside the sludge pellet, it induced the aerobic zone for nitrification at the pellet surface and the anaerobic zone for denitrification at the pellet core. Moreover, the use of entrapped sludge can provide advantages of higher mechanical strength and chemical resistance [19].

Internally present carbonaceous substrates (such as poly-*β*-hydroxybutyrate; PHB) have been used as the carbon source for denitrification for the famine period [20], especially in aerobic 1 stage of the ESR. When acetate was added in anaerobic 1 stage, a sharp decrease in NO_2_-N and NO_3_-N occurred and this is due to the denitrification from acetate consumption as the carbon source. Theoretically, the reduction of NO_2_-N and NO_3_-N values from 13 mg/L requires a COD of ~40 mg/L [21]. However, in this experiment the COD of 140–180 mg/L was consumed in anaerobic 1 stage and this suggests that some acetate present was utilised for PHB synthesis and this acted as the carbon source during the aerobic 1 stage. The nitrification and denitrification rates of the ESR were 0.3 and 0.1 mg/L-min, respectively, as summarised in Table 3. The ESR showed lower denitrification rate compared to the SSR and this is owing to the microorganisms located in the core of the pellet requiring the penetration of acetate from the surface.

Since the sludge pellet itself can provide aerobic and anaerobic zones for nitrification and denitrification, the effect of intermittent aeration on enhancing the ESR performance was investigated in this experimental setup. The ESR was operated under continuous aeration and the results are shown in Figure 3(a) (for days 26–40). The nitrogen removal efficiency decreased to 58.4% and the NO_3_-N content remained ~16.6 mg/L in the effluent. Ineffective denitrification was found to occur in the case of continuous aeration. The denitrification rate decreased to 0.05 mg/L-min while the nitrification rate increased slightly to 0.4 mg/L-min (not shown here). Continuous and intermittent aeration had a strong impact on the ratio of aerobic and anaerobic zones inside the pellet and this had a subsequent effect on the nitrification and denitrification activities. From these data, it can be concluded that two hours of intermittent aeration can enhance the ESR performance, with the highest efficiency being ~98.3% for a nitrification rate of 0.3 mg/L-min and a denitrification rate of 0.1 mg/L-min.

### 3.3. Performance of Mixed Sludge Reactor

As seen from the case of the previous two reactors, the nitrification rates were increased by increasing air flow rates or by continuous aeration; however this resulted in the lowering of the denitrification rate. To overcome this issue, the mixed sludge reactor was developed. This setup consists of a suspended sludge chamber with continuous aeration (aerobic) for nitrification and a sludge pellet chamber with no aeration (anaerobic) for denitrification. The DO in the aerobic and anaerobic stages was 4-5 and 3-4 mg/L, respectively. The results showed that the nitrogen removal efficiency was 45% and a high NO_3_-N content of 20.2 mg/L was found in the effluent. Ineffective denitrification was also found to occur in this reactor, since the DO of 3-4 mg/L was too high to produce a sufficiently large anaerobic zone inside the pellet. Moreover, the presence of excess acetate and oxygen in the anaerobic stage induced the growth of competitive heterotrophic microorganisms at the pellet surface [22]. These competitive microorganisms consumed some acetate resulting in insufficient acetate penetration for denitrification and PHB synthesis.

**Figure 4:**
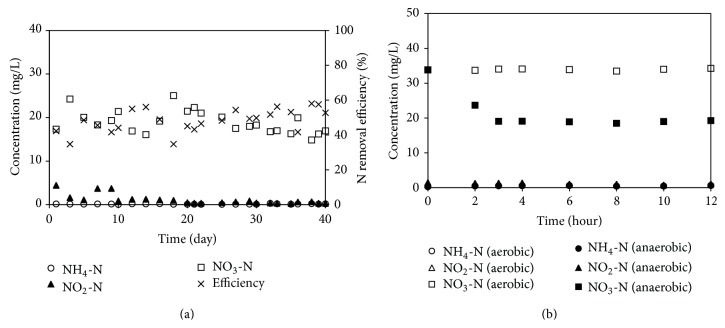
(a) Nitrogen removal efficiency and (b) nitrogen profile of MSR.

Since the competitive microorganisms had faster growth rate than the nitrifying and denitrifying microorganisms, the competitive microorganisms grew and became a suspended sludge in the anaerobic rector. At day 25, the concentration of the suspended sludge in the anaerobic reactor was ~100 mg/L. From Figure 4(b), the reduction of NH_4_-N from 40 mg/L to 0.5 mg/L and the increase in NO_3_-N to approximately 35 mg/L in the aerobic stage reveal that nitrification was the dominant process. In the meanwhile, the decrease in NO_3_-N in the anaerobic stage shows that only denitrification occurred. The nitrification and denitrification rates of the MSR were ~0.20 and 0.05 mg/L-min, respectively.

Furthermore, the C/N ratio of MSR operation was increased to 2.5 to enhance the denitrification activity. The results in Figure 4(a) (for days 26–30) show that the efficiency was ~50–60% which was similar to that seen for the MSR operating at a C/N ratio of 1.5. Moreover, the nitrification and denitrification rates were stable at 0.20 and 0.05 mg/L-min, respectively. Greater acetate addition did not enhance the denitrification activity, but it did increase the volume of suspended sludge in the anaerobic part. The highest efficiency of the reactor was 45% and the high DO in the anaerobic part was a major contributor towards the inefficient denitrification at the higher C/N ratio.

## 4. Conclusions

The performance of three reactors—suspended sludge reactor (SSR), entrapped sludge reactor (ESR), and mixed sludge reactor (MSR)—were assessed in terms of biological NH_4_-N removal from wastewater. The results demonstrated that the ESR achieved the highest nitrogen removal efficiency of 98.3%, while the efficiencies for SSR and MSR were ~64.7% and ~45.1%, respectively. The main process for NH_4_-N removal in all reactors was nitrification and denitrification. However, the significant advantages of ESR over the other reactors were (i) high denitrification rate of 1.8 mg/L-min by use of entrapped sludge and intermittent aeration and (ii) effective denitrification by use of internal and external carbon. Moreover, the cost of ESR setup including sludge entrapment and operation was comparable to the SSR and MSR setup.

## Figures and Tables

**Table 1:** Summary of biological nitrogen removal processes.

Process	Major reaction	Required atmospheric condition
Nitrification	NH_4_-N + O_2_→ NO_2_-N → NO_3_-N	Aerobic
Denitrification	NO_3_-N → N_2_	Anaerobic
SND	NH_4_-N + O_2_→ NO_3_-N → N_2_	Aerobic and anaerobic
SHARON	NH_4_-N + O_2_→ 0.5NH_4_-N + 0.5NO_2_-N	Aerobic (low O_2_)
Anammox	NH_4_-N + NO_2_-N → N_2_	Anaerobic

**Table 2:** Summary of conditions in the reactor.

Condition	Suspended sludge reactor (SSR)	Entrapped sludge reactor (ESR)	Mixed sludge reactor (MSR)
Working volume (L)	10	3	3
Influent feed	Batch	Batch	Continuous
Air supply	Intermittent	Intermittent	Continuous
Air flow rate (L/min)	0.5	0.2	0.2
Dissolved oxygen (DO) in aerobic condition (mg/L)	4-5	4-5	4-5
Dissolved oxygen (DO) in anaerobic condition (mg/L)	0.5	0.5	3-4
Influent NH_4_-N (mg/L)	40–50	40–50	40–50
Influent NO_2_-N (mg/L)	<3	<3	<3
Influent NO_3_-N (mg/L)	<1	<1	<1
C/N ratio	1.5	1.5	1.5

**Table 3:** Summary of reactors performance.

Parameters	Suspended sludge reactor (SSR)	Entrapped sludge reactor (ESR)	Mixed sludge reactor (MSR)
Nitrogen removal efficiency (%)	64.7	98.3	45.1
Effluent NH_4_-N (mg/L)	0.0	0.0	0.1
Effluent NO_2_-N (mg/L)	0.0	0.6	1.6
Effluent NO_3_-N (mg/L)	15.0	0.1	20.2
Nitrification rate (mg/L-min)	0.2	0.3	0.2
Denitrification rate (mg/L-min)	1.8	0.1	0.05
Internal carbon consumption	No	Yes	No
